# Maize/peanut intercropping improves nutrient uptake of side-row maize and system microbial community diversity

**DOI:** 10.1186/s12866-021-02425-6

**Published:** 2022-01-07

**Authors:** Xinhua Zhao, Qiqi Dong, Yi Han, Kezhao Zhang, Xiaolong Shi, Xu Yang, Yang Yuan, Dongying Zhou, Kai Wang, Xiaoguang Wang, Chunji Jiang, Xibo Liu, He Zhang, Zhimeng Zhang, Haiqiu Yu

**Affiliations:** 1 Peanut Research Institute, College of Agronomy, Shenyang Agricultural University, Shenyang, 110866 China; 2 Shandong Peanut Research Institute, Qingdao, 266100 Shandong China

**Keywords:** Maize, Peanut, Wide-strip intercropping, Nitrogen content, 16S/ITS, Soil enzyme

## Abstract

**Background:**

Intercropping, a diversified planting pattern, increases land use efficiency and farmland ecological diversity. We explored the changes in soil physicochemical properties, nutrient uptake and utilization, and microbial community composition in wide-strip intercropping of maize and peanut.

**Results:**

The results from three treatments, sole maize, sole peanut and intercropping of maize and peanut, showed that intercropped maize had a marginal advantage and that the nutrient content of roots, stems and grains in side-row maize was better than that in the middle row of intercropped maize and sole maize. The yield of intercropped maize was higher than that of sole cropping. The interaction between crops significantly increased soil peroxidase activity, and significantly decreased protease and dehydrogenase activities in intercropped maize and intercropped peanut. The diversity and richness of bacteria and fungi decreased in intercropped maize rhizosphere soil, whereas the richness of fungi increased intercropped peanut. *RB41*, *Candidatus-udaeobacter*, *Stropharia*, *Fusarium* and *Penicillium* were positively correlated with soil peroxidase activity, and negatively correlated with soil protease and dehydrogenase activities. In addition, intercropping enriched the functional diversity of the bacterial community and reduced pathogenic fungi.

**Conclusion:**

Intercropping changed the composition and diversity of the bacterial and fungal communities in rhizosphere soil, enriched beneficial microbes, increased the nitrogen content of intercropped maize and provided a scientific basis for promoting intercropping in northeastern China.

**Supplementary Information:**

The online version contains supplementary material available at 10.1186/s12866-021-02425-6.

## Background

Maize and peanut are major grain and oil crops and are important for ensuring food security in China. Sole cropping has been widely used in recent decades to facilitate planting, field management and mechanization. Sole cropping improved yield by increased fertilizer application; however, it was not only detrimental to grain production in China [1] but also disturbed the ecological stability of the soil microbial community and limited environmental sustainability [2]. Previous studies have found that wide-strip intercropping has the advantages of using marginal effects to increase yield, to optimize population structure, and to promote light energy utilization [3, 4]. This method is also suitable for mechanized seeding, fertilization and field management [5]. Maize and peanut strip intercropping not only improves crop yield and water and fertilizer utilization efficiency but also reduces competition for major soil nutrients, increases beneficial soil microorganism numbers and diversity, and reduces pathogenic and poisonous microorganisms, effectively improving the ecological environment of farmland [6, 7] while helping to reduce carbon emissions and to increase the economic value of the ecosystem [8].

Previous studies have shown that crop nutrient uptake was affected by soil nutrient distribution and neighbouring crops in an intercropping system [9]. According to a report, the intercropping of proso millet and mung bean has increased the nitrogen absorption efficiency by 96 and 71.6%, respectively, on the Loess Plateau of China, due to the complementarity of crops [10]. The maize grain nitrogen uptake was increased by 25.5% in strip intercropping of maize and soybean in southwestern of China [11]. Therefore, by changing the spatial distribution of roots, delaying root senescence and increasing root activity to achieve niche complementation, the nitrogen uptake and utilization of maize can be improved [12, 13]. Legumes have a symbiotic relationship with nitrogen-fixing bacteria and through increased abundance of the nitrogen-fixing gene *nifH*, leguminous crops are able to obtain nitrogen from the air; therefore, intercropping with legumes allows neighbouring crops to absorb more nitrogen from the soil [14–16]. In addition, through interactions between crops, the acquisition of soil resources is improved. Studies have shown that maize root exudates promote the expression of chalcone-flavanone isomerase and the synthesis of flavonoids in bean roots, increasing nodulation and nitrogen fixation [17–19].

Intercropping also results in indirect promotion by changing the soil microbial community composition and affecting nutrient transport and mineralization [13, 20]. Previous studies have found that intercropping changes the composition and function of microbial communities. The abundance of the nitrogen-fixing microbes *Rhizobium hainanense*, *R. leguminosarum* and *Frankia* spp. was promoted in the rhizosphere of peanut when intercropped with maize [16]. Some studies have also shown that root exudates affect the composition and function of the rhizosphere microbial community and promote soil organic matter mineralization and the nitrogen cycle [21]. Intercropping cassava and peanut induced ethylene release resulted in an increase in the abundance of *Actinomycetes* in the rhizosphere of peanut and promoted the absorption of soil available nutrients, thus increasing the yield of peanut [22]. In a study on the intercropping of maize and faba bean, maize root exudates increased nodule formation and biological nitrogen fixation in faba bean roots, and flavonoids in leguminous root exudates stimulated *NOD* gene expression in rhizobia [19]. Therefore, the relationship between microorganisms, crops and soil under maize and peanut intercropping reveals the adaptation of crops to the microbial environment and helps to understand the specific root exudates and signal substances caused by changes in the soil microbial composition [23], which help protect the ecological environment and develop sustainable agriculture.

Northeastern China has a temperate monsoon climate, drought and relatively low rainfall during the crop growing season, and there has been limited systematic research into the characteristics and mechanism of nitrogen uptake by crops and the correlation between soil physicochemical properties and soil microorganisms. A field experiment was conducted in this study, the purpose of what 1) determine the changes in the structure composition and diversity of the bacterial and fungal communities at the genus level under intercropping, 2) determine whether there is a correlation between soil enzyme activities and the bacterial and fungal communities, and 3) identify the mechanisms underlying yield increases from the perspective of the diversity of soil microorganisms to maintain the balance of the soil ecosystem and increase productivity through sustainable agriculture.

## Results

### Effect of intercropping on the nitrogen content and yield of maize and peanut

**Fig. 1:**
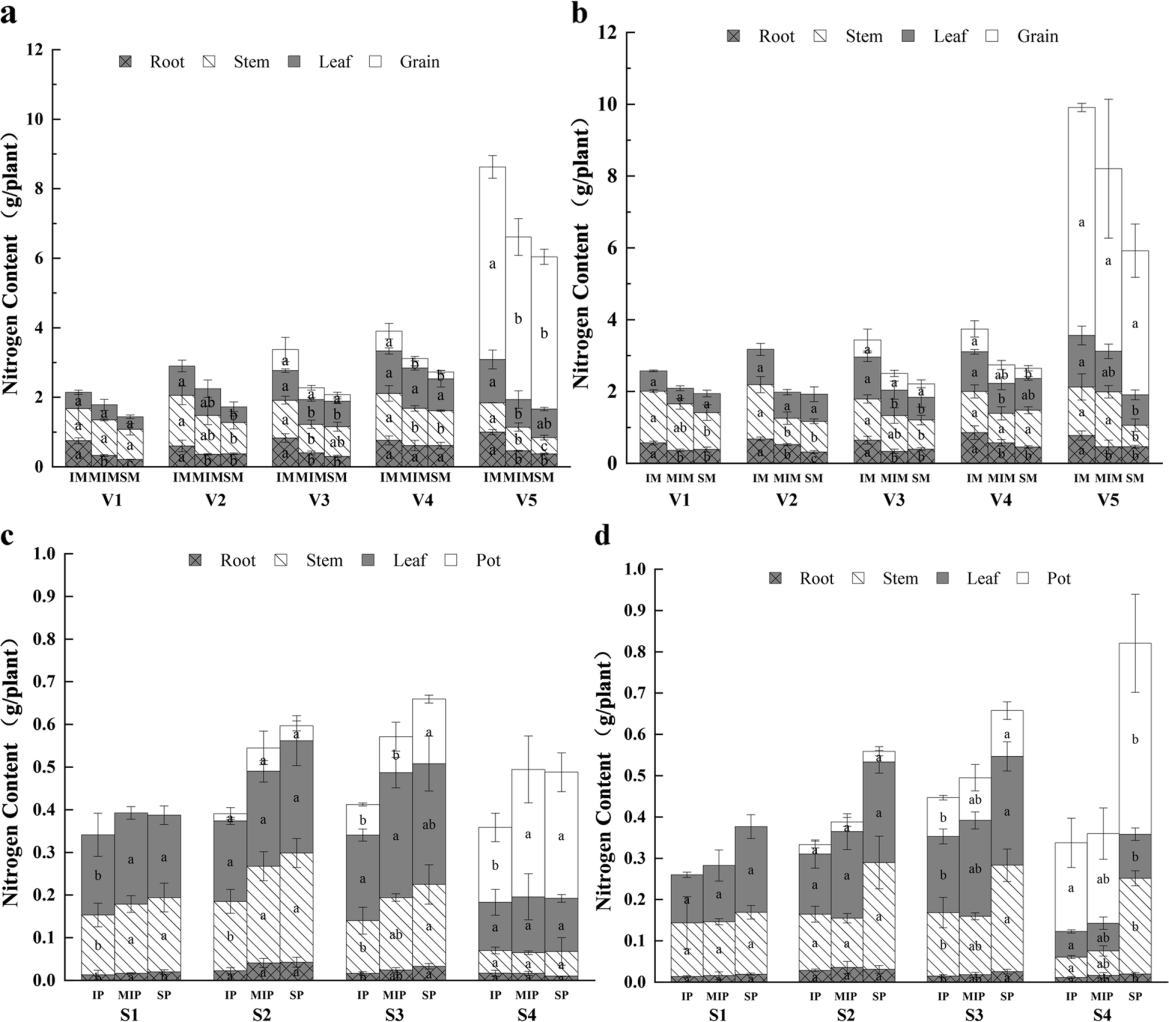
Nitrogen content of crops under intercropping of maize and peanut. **a** The nitrogen content in various organs of maize in 2018, **b** The nitrogen content in various organs of maize in 2019, **c** The nitrogen content in various organs of peanut in 2018, **d** The nitrogen content in various organs of peanut in 2019. V1: trumpeting stage, V2: heading stage, V3: anthesis and silking stage, V4: grain-filling stage, V5: mature stage, S1: seedling stage, S2: flowering stage, S3: podding stage, S4: mature stage. SM: sole maize, MIM: the middle row of intercropped maize, IM: intercropped maize, SP: sole peanut, MIP: the middle row of intercropped peanut, IP: intercropped peanut

Changes in the nitrogen content of maize and peanut were similar between 2018 and 2019 (Fig. 1). The nitrogen contents of maize followed order intercropped maize (IM) > sole maize (SM), intercropped maize (IM) > the middle row of intercropped maize (MIM). The roots of maize (IM) were clearly higher than those of sole maize (SM) (Fig. 1a, b), indicating that intercropped maize had a marginal advantage, and the nitrogen content in maize was increased. The nitrogen contents of peanut followed the order intercropped peanut (IP) < sole peanut (SP), intercropped peanut (IP) < the middle row of intercropped peanut (MIP), and the stems and leaves of intercropped peanut were significantly lower than those of sole peanut (Fig. 1c, d). In addition, the ear length and number of grains per spike significantly affected the maize yield. Compared with sole maize, the yield of intercropped maize significantly increased, by 30.34% (2018) and 24.8% (2019) (Table S1). Compared with sole peanut, the yield of intercropped peanut decreased, by 33.49% (2018) and 2.4% (2019), and the 100-kernel weight significantly affected the peanut yield (Table S1).

### Effect of intercropping on the soil TN content and soil enzyme activities of maize and peanut

The soil TN contents of sole maize (SM) and sole peanut (SP) were significantly higher than those of intercropped maize (IM) and intercropped peanut (IP) (Table 1), showing that intercropping increased soil nutrient consumption. The soil TN contents of intercropped maize (IM) and intercropped peanut (IP) was lower than those of the middle row of intercropped maize (MIM) and the middle row of intercropped peanut (MIP), respectively (Table 1). The TN content of the shared soil of intercropped maize and peanut (II) between the intercropped maize (IM) and intercropped peanut (IP) was not significantly different (Table S2). It was speculated that the interspecific root interaction between intercropped maize (IM) and intercropped peanut (IP) promoted soil nutrient uptake and utilization.

**Table 1:** Soil total nitrogen (TN) content of different soil samples (mg/kg)

Stage	V1	V2/S1	V3/ S2	V4/ S3	V5/S4
Sample					
SM	235.43 ± 1.59a	224.44 ± 2.66a	186.01 ± 2.56a	157 ± 0.85a	125.00 ± 1.18a
MIM	4.34 ± 0.89c	6.34 ± 0.33b	5.19 ± 0.09b	6.59 ± 0.50b	2.91 ± 0.49b
IM	7.39 ± 0.15b	6.63 ± 0.37b	5.62 ± 0.19b	4.93 ± 0.54c	2.31 ± 0.70b
SP	258.16 ± 1.42a	217.66 ± 1.62a	179.37 ± 1.37a	133.91 ± 1.36a	85.93 ± 0.61a
MIP	5.87 ± 0.68b	7.73 ± 0.16b	6.50 ± 0.17b	11.47 ± 0.53b	2.31 ± 0.65b
IP	6.98 ± 0.50b	6.40 ± 0.31b	7.13 ± 0.22b	9.67 ± 0.29b	3.38 ± 0.05b

Note: V1: trumpeting stage, V2: heading stage, V3: anthesis and silking stage, V4: grain-filling stage, V5: mature stage, S1: seedling stage, S2: flowering stage, S3: podding stage, S4: mature stage, SM: sole maize, MIM: the middle row of intercropped maize, IM: intercropped maize, SP: sole peanut, MIP: the middle row of intercropped peanut, IP: intercropped peanut. Different letters indicate significant differences at 0.05

**Fig. 2:**
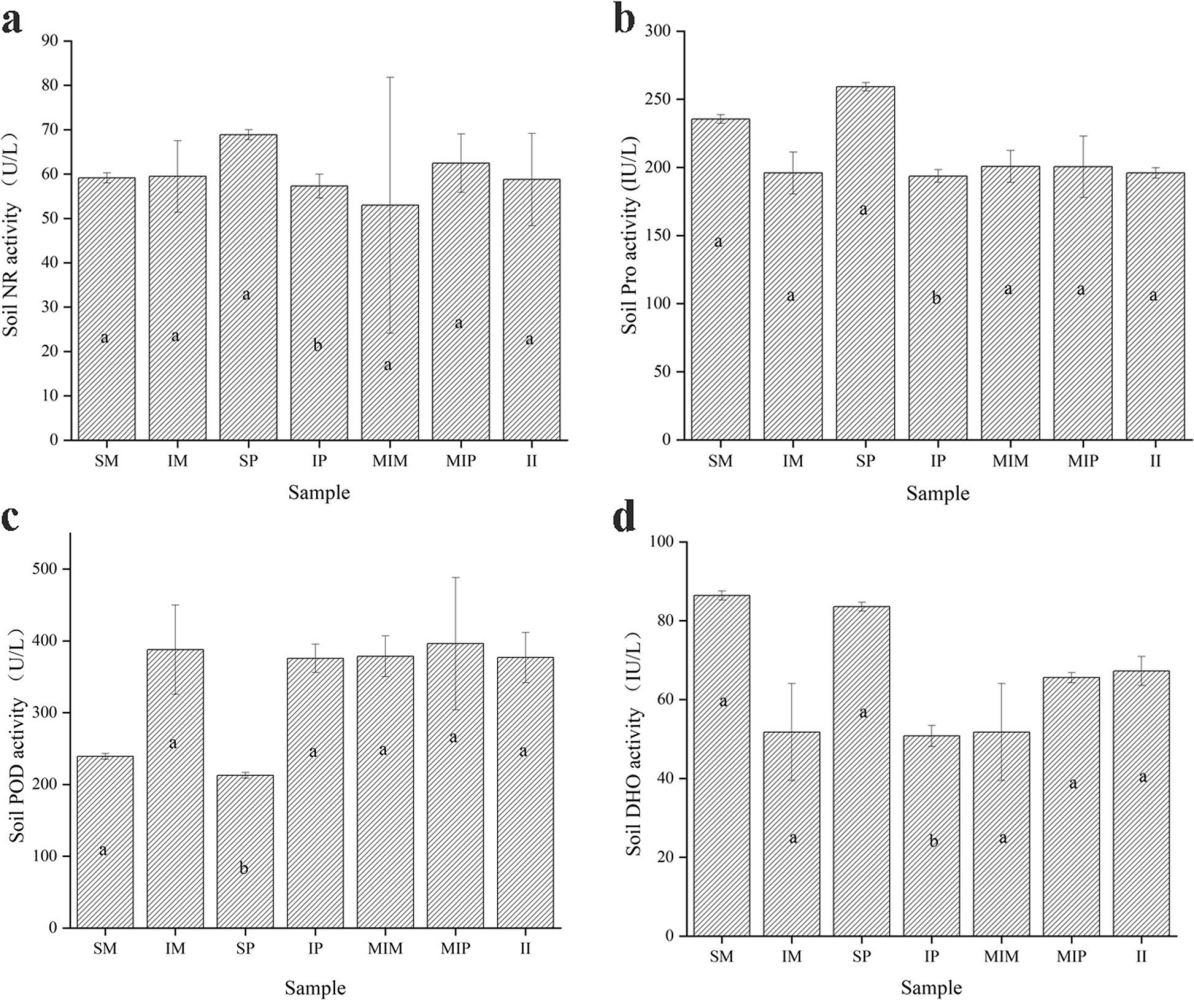
Changes in soil enzyme activities under intercropping of maize and peanut. **a** Soil NR activity, **b** Soil Pro activity, **c** Soil POD activity, **d** Soil DHO activity, SM: sole maize, MIM: the middle row of intercropped maize, IM: intercropped maize, SP: sole peanut, MIP: the middle row of intercropped peanut, IP: intercropped peanut, II: the shared soil of intercropped maize and peanut

Compared with sole maize (SM), the activity of peroxidase (POD) (Duncan test, *P* < 0.05) in intercropped maize (IM) increased (Fig. 2), and the activities of protease (Pro) (Duncan test, *P* < 0.05) and dehydrogenase (DHO) decreased (Fig. 2b, d). The POD in intercropped peanut (IP) soil was increased (Fig. 2c), and the activities of Pro (Duncan test, P < 0.05) and DHO (Duncan test, P < 0.05) decreased compared with those of peanut alone (Fig. 2c, d). Compared with the middle row of intercropping, the change in the soil enzyme activities in the side row was similar (Fig. 2). Compared with cropping alone, the intercropping of maize and peanut significantly increased the activity of POD, and decreased the activities of Pro and DHO. Furthermore, the activities of the four enzymes were not significant different among intercropped maize (IM), intercropped peanut (IP) and the shared soil of intercropped maize and peanut (II) (Table S3). Correlation analysis showed that POD activity was significantly negatively correlated with TN, while the activities of other enzyme were significantly positively correlated with TN (Fig. S1).

### OTUs and diversity of the rhizosphere soil microbial community

**Fig. 3:**
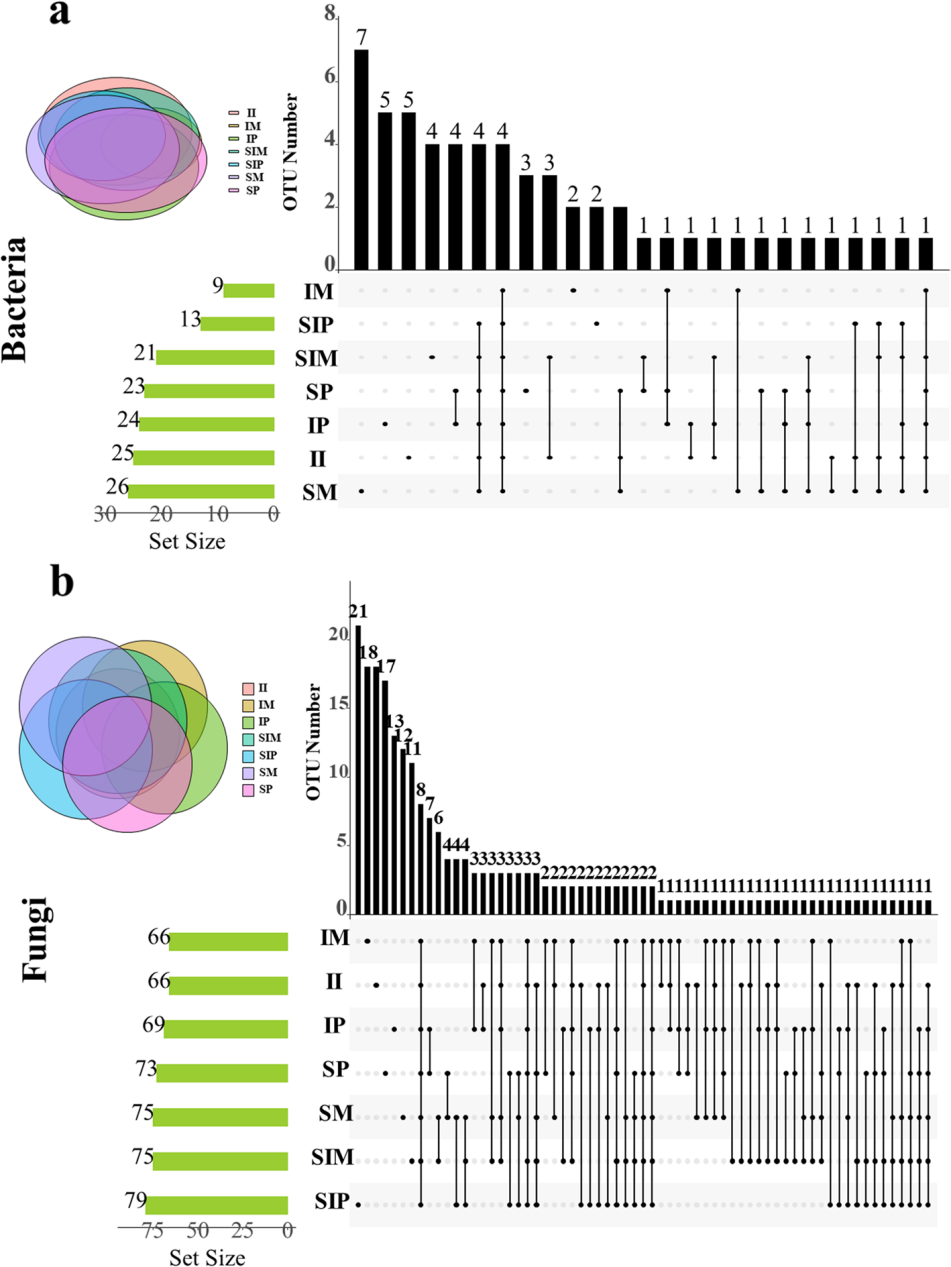
UpSet diagram of the distribution of OTUs in the microbial community. **a** Bacterial community, **b** Fungal community, SM: sole maize, SIM: the shared soil of sole maize, IM: intercropped maize, SP: sole peanut, SIP: the shared soil of sole peanut, IP: intercropped peanut, II: the shared soil of intercropped maize and peanut

Compared with sole maize (SM), the OTUs of bacteria and fungi in intercropped maize (IM) decreased markedly by 9.7 and 10.4%, respectively. However, compared with sole peanut (SP), the variation in the OTUs in intercropped peanut (IP) was different. The number of OTUs was lower (by 3.9%) in the bacterial community, while the number of fungal OTUs was higher (by 7.9%) (Fig. S2). Through UpSet diagram analysis of OTUs, common microorganisms were found in different samples. Bacterial OTU analysis indicated that 4 OTUs were common to all samples. Four OTUs were shared by intercropped peanut (IP) and sole peanut (SP) compared with 1 OTU shared by intercropped maize (IM) and sole maize (SM). Three OTUs were shared by the shared soil of sole maize (SIM) and the shared soil of intercropped maize and peanut (II) (Fig. 3a, Table S4). Fungal OTU analysis indicated that 8 OTUs were shared by all samples. Seven OTUs were shared by intercropped peanut (IP) and sole peanut (SP). Two OTUs were shared by the shared soil of sole peanut (SIP) and the shared soil of intercropped maize and peanut (II) was higher than that of 1 OTUs shared by the shared soil of sole maize (SIM) and the shared soil of intercropped maize and peanut (II) (Fig. 3b, Table S4).

**Fig. 4:**
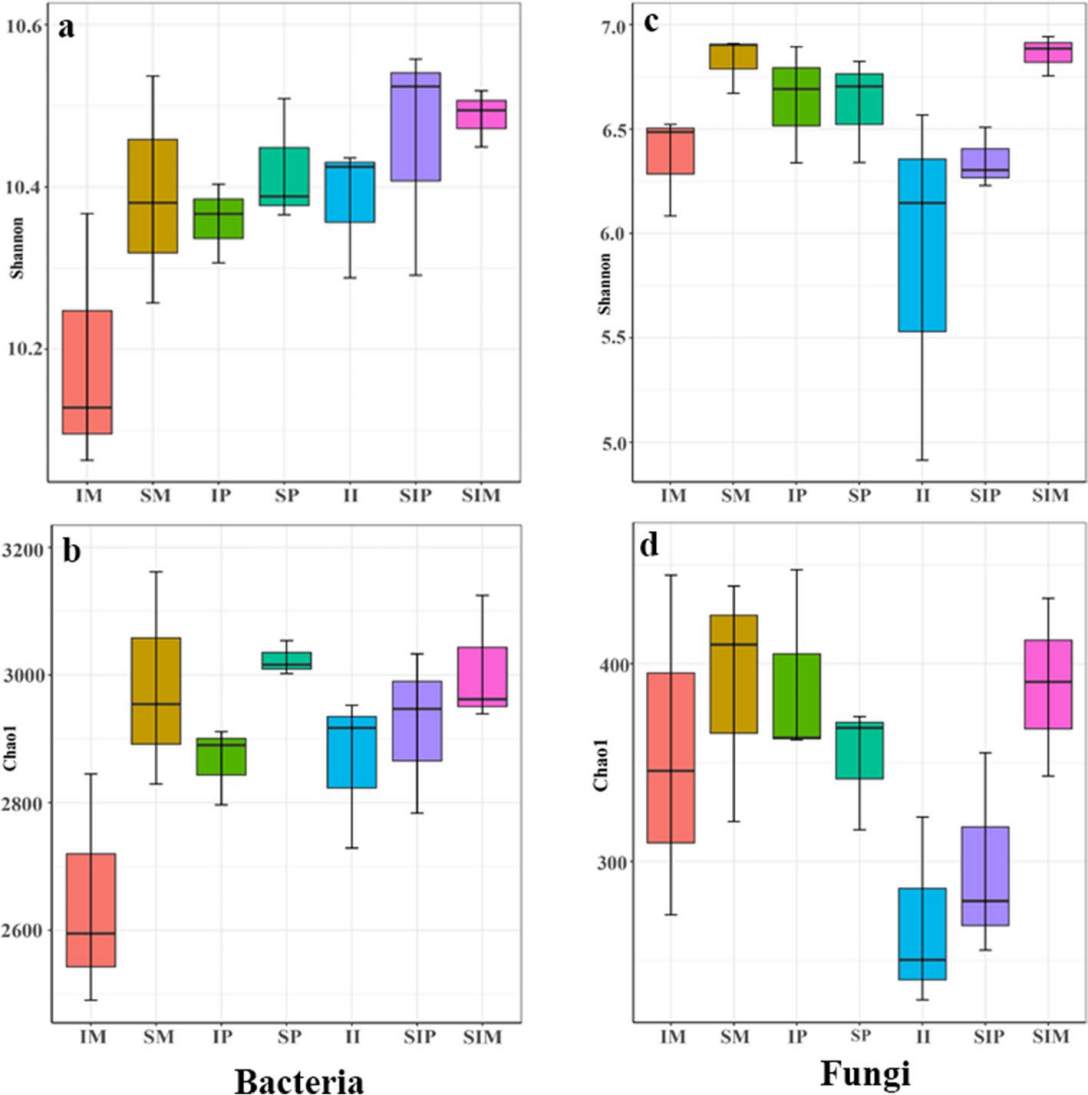
Diversity and richness of the microbial community**. a**, **b** Bacterial community, **c**, **d** Fungal community, SM: sole maize, SIM: the shared soil of sole maize, IM: intercropped maize, SP: sole peanut, SIP: the shared soil of sole peanut, IP: intercropped peanut, II: the shared soil of intercropped maize and peanut

The diversity and richness of bacteria and fungi in intercropped maize (IM) were lower than those in sole maize (SM), and those in intercropped peanut (IP) were lower than those in sole peanut (SP), but the diversity and richness of fungi were increased. The bacterial and fungal diversity and richness of the shared soil of intercropped maize and peanut (II) were lower than those of the shared soil of sole peanut (SIP) and the shared soil of sole maize (SIM) (Fig. 4).

### Analysis of the microbial community composition

**Fig. 5:**
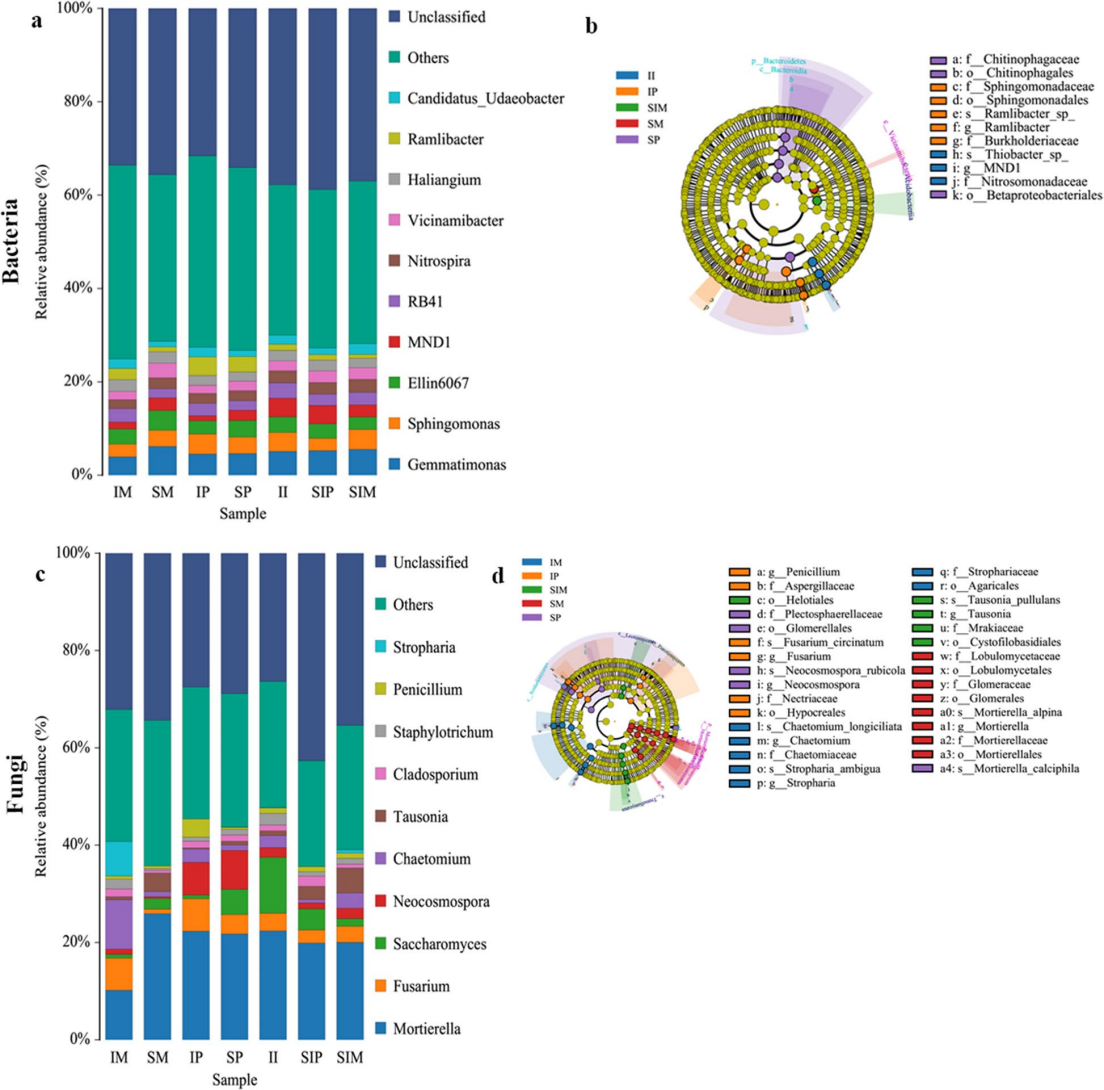
The relative abundance of the microbial community**. a**, **b** Bacterial community, **c**, **d** Fungal community. The histogram of the distribution figures shows the relative abundance of the top 10 rhizosphere soil bacteria and fungi at the genus level. A colour represents a species, and the length of the colour block represents the relative abundance ratio of species at the genus level. The circle radiating from the inside to the outside of the branch diagram represents the classification level from phylum to species; each small circle at different classification levels represents a classification at that level, and the diameter of the small circle corresponds to the relative abundance. Different colours indicate different groups, and nodes of different colours indicate the groups of microorganisms that play an important role in the groups represented by the colours

Although the interaction between crops did not increase the diversity and richness of the bacterial and fungal communities, it increased the abundance of some bacteria and fungi. In the bacterial community, compared with sole maize (SM) and sole peanut (SP), the relative abundances of *RB41*, *Haliangium*, *Ramlibacter*, *Candidatus-Udaeobacter* and *Sphingomonas* were higher in intercropped maize (IM) and intercropped peanut (IP) (Fig. 5a, Table S5). Compared with the shared soil of sole peanut (SIP) and the shared soil of sole maize (SIM), the relative abundance of *Ellin6067*, *MND1*, *RB41 and Ramlibacter* increased in the shared soil of intercropped maize and peanut (II), whereas the relative abundance of *Gemmatimonas* decreased (Fig. 5a, Table S5). To identify the representative microbes in the samples, soil microbes from the different treatments were compared using LEfSe analysis. At the bacterial genus level, *Ramlibacter* and *MND1* were significantly enriched in intercropped peanut (IP) and the shared soil of intercropped maize and peanut (II) (Fig. 5b). In the fungal community, compared with sole maize (SM) and sole peanut (SP), the relative abundances of *Fusarium*, *Chaetomium*, *Cladosporium* and *Penicillium* were higher in intercropped maize (IM) and intercropped peanut (IP) (Fig. 5c, Table S5). The relative abundances of *Neocosmospora* and *Staphylotrichum* increased in intercropped maize (IM), but decreased in intercropped peanut (IP) (Fig. 5c, Table S5). The relative abundance of *Mortierella*, *Fusarium, Staphylotrichum* and *Penicillium* increased in the shared soil of intercropped maize and peanut (II), whereas *Tausonia* decreased (Fig. 5c, Table S5)*.* At the fungal genus level, the abundance of *Chaetomium* was significantly enriched in intercropped maize (IM), and *Penicillium* and *Fusarium* were significantly enriched in intercropped peanut (IP) (Fig. 5d).

### Analysis of the association between the bacterial/fungal community and soil physicochemical properties

**Fig. 6:**
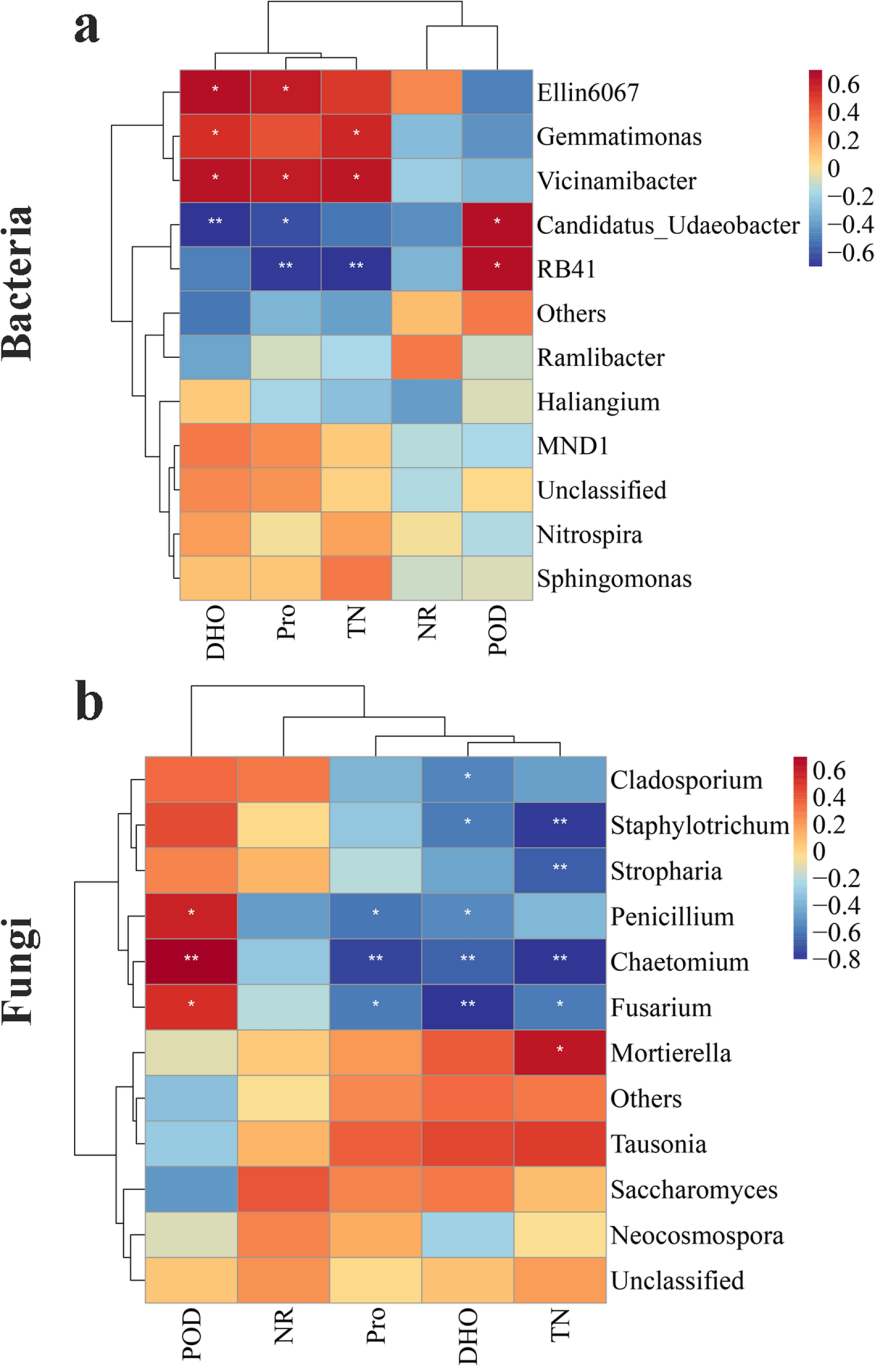
Cluster heatmap of the correlation between the soil physiochemical properties and microbial community. **a** Bacterial community, **b** Fungal community

In the bacterial community, *RB41 and Candidatus-Udaeobacter* were significantly positively correlated with POD activity and negatively correlated with TN, Pro and DHO. *Vicinamibacter* and *Gemmatimonas* were significantly positively correlated with TN, Pro and DHO (Fig. 6a). In the fungal community, *Chaetomium, Fusarium* and *Penicillium* were extremely significantly and significantly positively correlated with POD activity, and extremely significantly and significantly negatively correlated with DHO, Pro and TN. *Stropharia*, *Staphylotrichum* and *Cladosporium* were extremely significantly and significantly negatively correlated with TN and DHO, respectively (Fig. 6b).

### Analysis of bacterial/fungal community functional prediction

**Fig. 7:**
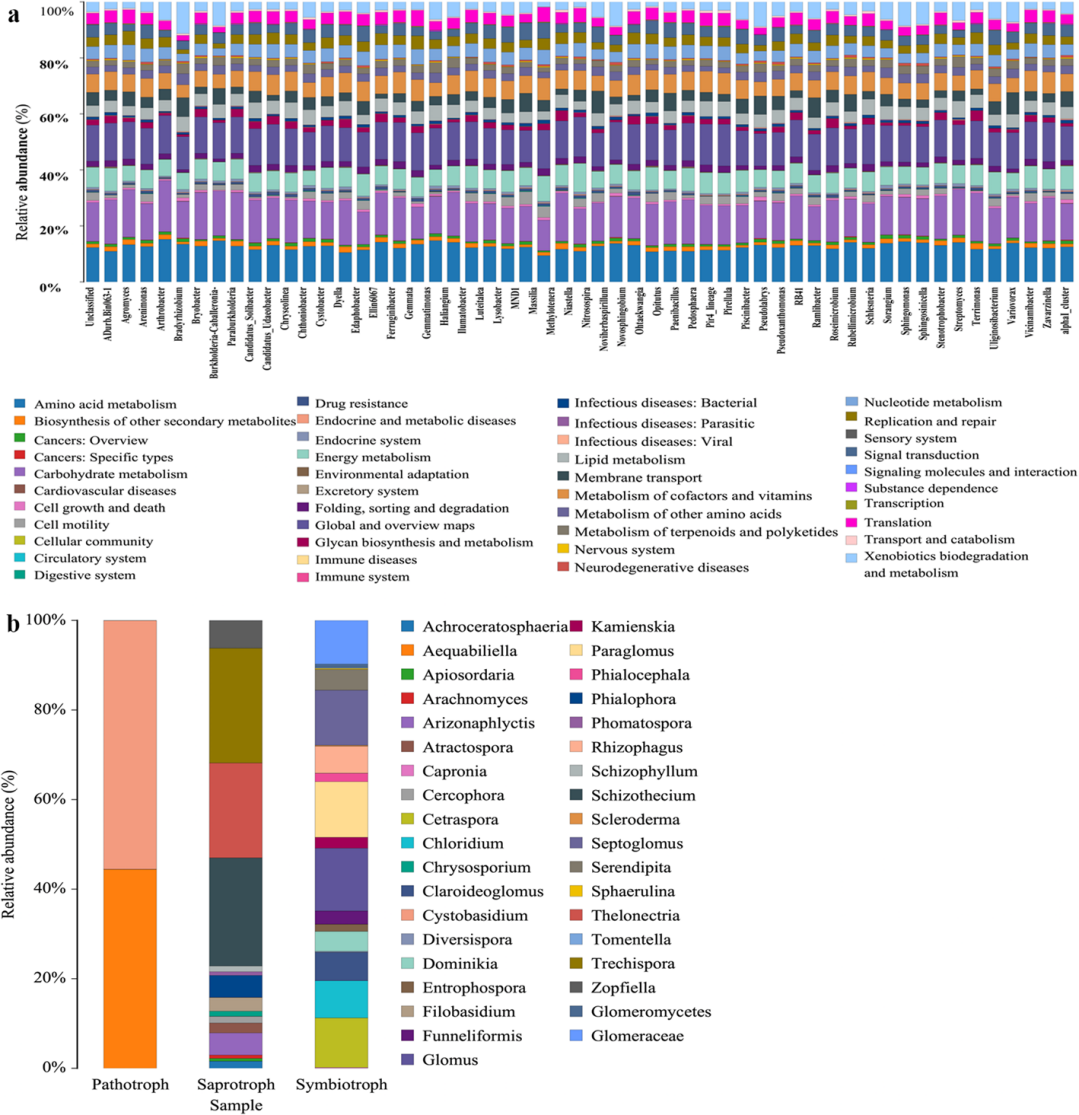
The Predictions of function in the bacterial community (**a**), and fungal community (**b**)

Analysis of the KEGG pathways revealed that the functions of the first level of bacteria was mainly metabolism (Fig. S3). The functions of the second level, which included amino acid metabolism, carbohydrate metabolism and other amino acid metabolism accounted for a higher relative abundance (Fig. 7a). In the fungal community, intercropping of maize and peanut reduced the functional groups of pathogenic fungi in the soil, enriched the functional groups of saprotroph fungi (Fig. 7b), and improved the soil microecological environment.

### Discussion

Nitrogen is an essential element for plant growth and development and is the element most closely related to yield. In a gramineous and leguminous intercropping system, nitrogen content and yield in a gramineous crop were reported to be clearly promoted; thus, the land productivity improved [24]. In the current study, maize had a marginal advantage in the intercropping of maize and peanut. The nitrogen content in the roots, stems and leaves of the side row of intercropped maize (IM) was significantly higher than that of the sole maize (SM) and the middle row of intercropped maize (MIM) (Fig. 1a, b), which is consistent with the findings of another study [4]. Due to the adjustment of root length density and root distribution, the nitrogen uptake per unit root length increased, compared with that of sole cropping [25].Maize competes strongly for nitrogen and absorbs more nitrogen than peanut, so the nitrogen content in intercropped maize (IM) significantly increased and the yield of maize was promoted [26]. In the middle and late growth periods, the intensified shading of intercropped maize (IM) reduced the photosynthesis of intercropped peanut (IP), which further affected the absorption of nutrients by the peanut (Fig. 1c, d). In intercropped maize and soybean, light transmittance increased after defoliation of the top two leaves of maize, and the nitrogen absorption of soybean increased by 5% (grain), 10% (stem) and 14% (root) [27]. The shading caused by maize inhibited the growth of peanut, and the yield of intercropped peanut (IP) decreased (Table S1). The ear length, number of grains per spike and 100-kernel weight were the main yield components (Table S1), which were the same as the findings of a previous study [4].

In this study, the interaction between intercropped maize (IM) and intercropped peanut (IP) promoted soil nutrient uptake and utilization (Table 1). The results are consistent with previous findings, i.e., a decrease in soil TN in intercropped Chinese milk vetch and rape [28]. According to the report, the root density distribution was different under different soil depths, which affected the absorption and utilization of soil nutrients [29]. Moreover, the interaction or competition between crops maintained the basic stability of the soil physicochemical properties (Table S2–3) [13, 30].

Soil microorganisms are one of the main sources of soil enzymes, and there is a correlation between soil enzyme activity and microorganisms [11, 31]. POD is involved in the degradation of hydrocarbons and their intermediates [32]. In this study, intercropped maize (IM) and intercropped peanut (IP) caused a significant increase in POD activity (Fig. 2c). This was because the interaction between crops affected the soil enzyme activity and nutrient cycling process by affecting the activity and abundance of microorganisms [32]. Pro is involved in the conversion of amino acids and other nitrogen containing organic compounds present in soil, and its hydrolysates are one of the nitrogen sources for higher plants [33]. The Pro activity of intercropping maize (IM) and intercropping peanut (IP) showed a significant decrease (Fig. 2b). The inhibition of Pro was also observed in another study with intercropped sugarcane and peanut [33]. Soil DHO participates in the soil carbon cycle and promotes the dehydrogenation of carbohydrates and organic acids [34], which is in accordance with the findings of another study [35]. These results showed that the interaction between crops caused the soil POD activity to increase and the Pro and DHO activities to decrease. The soil Pro and DHO activities were positively correlated with TN (Fig. S1), which is consistent with previous studies, and changes in soil enzyme activity can change soil nutrients [34].

**Fig. 8:**
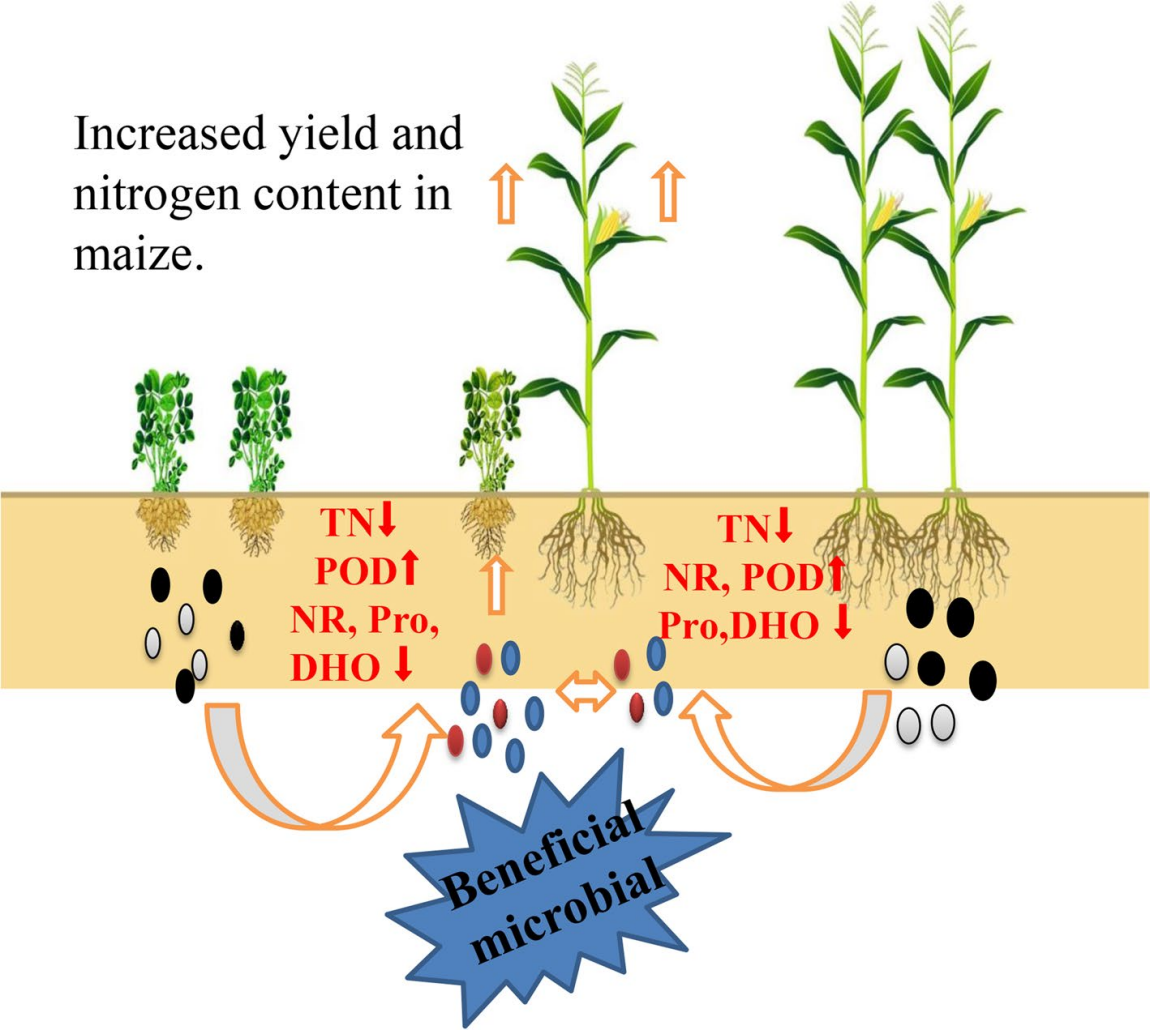
Overview of the promoting nutrient uptake by the interaction between maize and peanut

**Fig. 9:**
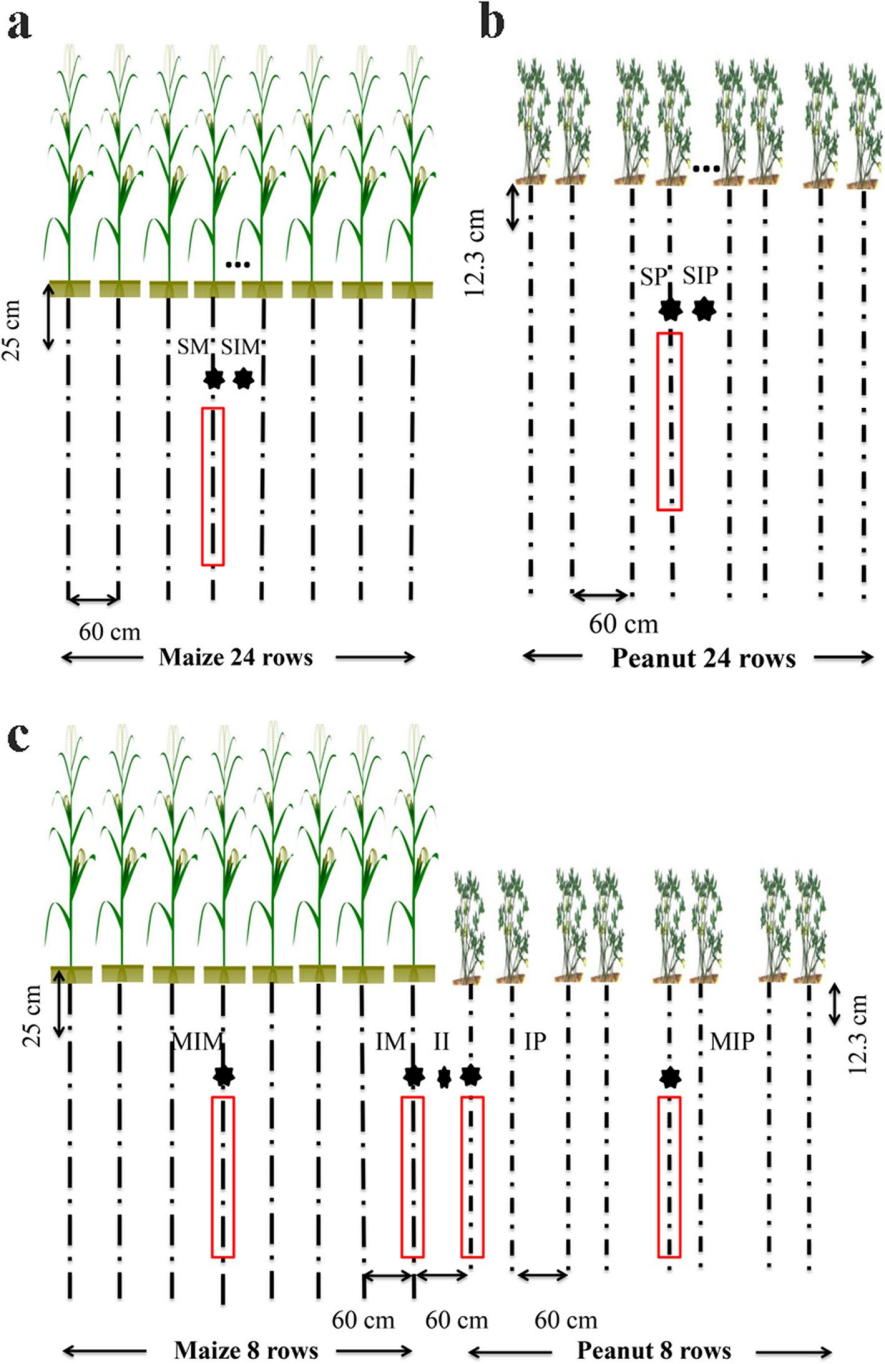
Plant patterns and sampling points for the field experiment**. a** Sole maize; **b** Sole peanut; **c** Intercropping of maize and peanut; ■ plant number per hole;  soil sample points; □ plant sample area. SM: sole maize, SIM: the shared soil of sole maize, MIM: the middle row of intercropped maize, IM: intercropped maize, SP: sole peanut, SIP: the shared soil of sole peanut, MIP: the middle row of intercropped peanut, IP: intercropped peanut, II: the shared soil of intercropped maize and peanut

Soil microorganisms play a key role in soil nutrient cycling and crop nutrient uptake [20]. We found that the diversity and richness of the bacterial community decreased in intercropped maize (IM) and intercropped peanut (IP) (Fig. 4a, b), which further verified that interactions between crops affect microbial diversity (Fig. 8) [13]. The relative abundances of *RB41* and *Ramlibacter* increased in intercropped maize (IM), intercropped peanut (IP) and the shared soil of intercropped maize and peanut (II) (Fig. 5a, Table S5, Fig. 9)*,* and intercropped peanut (IP) had a significantly higher abundance of *Ramlibacter* (Fig. 5b)*. Ramlibacter* belonging to Proteobacteria, comprises an enormous range of metabolic diversity [29], which is consistent with the findings that Proteobacteria are dominant bacteria [11]. In addition, *Sphingomonas* was also increased in intercropped peanut (IP) compared with sole peanut (SP) (Fig. 5a, Table S5), which has the characteristic of promoting nitrogen fixation and dehydrogenation [36], thus enhancing the uptake of nutrients in the rhizosphere, improving the rhizosphere soil environment of intercropped peanut (IP), and maintaining the soil nitrogen balance. Hence, the interaction between crops improved the bacterial community composition and increased the abundance of beneficial bacteria (Fig. 8). The changes in the activity of soil enzymes indicated changes in microbial activity. The soil enzyme activities of POD, Pro and DHO were positively correlated with most bacteria (Fig. 6a) and negatively correlated with most fungi (Fig. 6b). The reason is that there are many kinds of microbes and the correlation between soil enzyme activity and bacteria/fungi was not specific or unique. A single bacterium can affect a variety of enzyme activities, so it is necessary to further explore or study the functional properties of each bacterium and the mechanism of soil enzyme activities themselves [37]. The transport and metabolism of amino acids, carbohydrate transport and metabolism, and the metabolism of other amino acids involved in secondary functions were comparatively high, which is consistent with previous findings [33, 35]. A series of materials are produced when soil microorganisms participate in amino acid metabolism and carbohydrate metabolism. When the materials are perceived as signals by plants, they stimulate plant enzyme activity or cause changes in gene expression to ensure the survival of the bacteria, and then plant physiological metabolism and nutrient accumulation levels are adjusted to promote plant growth and development [38, 39].

Soil fungi decompose organic matter in crop residues and fertilizers [37]. In this study, the diversity and richness of the fungal community increased in intercropping peanut (IP) (Figs. 4 and 8), consistent with the promotion of fungal community growth under intercropping [40]. On the one hand, many biotic and abiotic factors can alter the fungal community, such as soil chemical properties, plant functional diversities and management practices. On the other hand, the variety and quantity of root exudates affect the abundance of the fungal community due to different crops [41, 42]. The relative abundances of *Mortierella, Fusarium, Chaetomium* and *Cladosporium* increased in intercropped maize (IM), intercropped peanut (IP) and the shared soil of intercropped maize and peanut (II) (Fig. 5c, Table S5, Fig. 8), and intercropped maize (IM) had a significantly higher abundance of *Chaetomium* (Fig. 5d). Intercropped peanut (IP) had a significantly higher abundance of *Fusarium* and *Penicillium* (Fig. 5d). *Chaetomium* is a beneficial fungus with biocontrol effects and is antagonistic to soil pathogenic bacteria [43]. *Mortierella* decomposes organic matter and promotes mineral uptake by plant roots. It also has the potential to secrete antimicrobials that inhibit pathogenic bacteria such as *Fusarium* [44–46]. Overall, the interaction between crops optimized the fungal community composition (Fig. 8). This was because different crops release chemical substances to the surrounding environment through allelopathy produced by secondary substances under intercropping. Another reason is that the soil physicochemical properties are related. Saprotrophic fungi were concentrated in rhizosphere soil as a dominant functional group and obtained nutrients by degrading dead host cells (Fig. 7b). These fungi are closely involved in the decomposition of organic matter and nutrients and can also produce a series of hydrolases and oxidases, which contribute to the decomposition of carbohydrates and increase the nutrients in soil organic matter [47]. Compared with sole cropping, the intercropped microecological environment was complex and microorganisms and plants were interdependent. These characteristics provides a theoretical basis for further understanding of the mechanism of plant nutrient absorption. Our results indicated that the staggered superposition of roots and the secretion of secondary metabolites among root systems under intercropping promoted the reproduction of rhizosphere fungi and improved microbial diversity [14]. In this regard, managing rhizosphere microbes and maintaining the balance of the soil microbial community assist plant growth and nutrient uptake.

### Conclusion

Our study clearly illustrated the mechanism underlying increased nutrient uptake from the perspective of microbial community diversity in intercropping of maize and peanut. Intercropping of maize and peanut increased soil POD activity, decreased soil Pro and DHO activities, and affected the composition of soil bacteria and fungi. The relative abundances of beneficial microbial *RB41*, *Candidatus-Udaeobacter*, *Chaetomium*, and *Mortierella* increased, which improved the microbial community composition. Therefore, the bacterial community functions of amino acid metabolism and carbohydrate metabolism were activated, and the groups of pathogenic fungi were reduced. Overall, the intercropping of maize and peanut stimulated soil microbial communities that were beneficial to plant growth and are appropriate in agricultural practice.

## Methods

### Field sites and experimental design

Field experiments were conducted in 2018 and 2019 in long-term plots at Shenyang Agricultural University, Shenyang, China (41°82′ N, 123°56′ E), which has a temperate monsoon climate with an average temperature of 8.4 °C and annual precipitation between 680 mm and 530 mm. The field site was previously used for sole peanut, and the soil was a brown loam (Table S6). The maize was hybrid Liang-yu 99 (*Zea mays* L.), semicompact plant with high nutrient efficiency (Dandong Denghai Seed Industry Co. Ltd., China). The peanut variety was Nong-hua 9 (*Arachis hypogaea* L.), which is upright and sparsely branched, with strong shade resistance and good comprehensive resistance (Peanut Research Institute of Shenyang Agricultural University, China). We used a single-factor randomized block design with three treatments comprising sole maize (SM), sole peanut (SP) and intercropping of maize and peanut (IMP) and included three replicate plots. We used a planting pattern of 8:8 wide belts to intercrop maize and peanut, and the crop rows were oriented north–south (Fig. 9). Basal fertilizer was applied before sowing; intercropping maize received with conventional compound fertilizer, i.e., 750 kg/hm^2^ (N-P_2_O_5_-K_2_O = 27–13-15) and peanut received potassium phosphate compound fertilizer i.e., 750 kg/hm^2^ (N-P_2_O_5_-K_2_O = 14–16-15) (Table S7). The sole maize and peanut plots consisted of 24 rows, and the plant density and fertilizer were the same as those used in the intercropping pattern. Intercropping maize was grown at a row distance of 60 cm, and the plant distance within a row was 25 cm, resulting in a density of 66,670 plants/hm^2^. Intercropping peanut was grown on a small ridge in double rows with a row distance of 60 cm, and the plant distance within a row was 12.3 cm, resulting in a density of 135,508 plants/hm^2^ (Fig. 9). The sowing and harvest dates of maize and peanut are shown in Supplementary Table S8. Other cultivation management measures were consistent with conventional field production.

### Sample collection

#### Plant samples

Maize (V1: trumpeting stage, V2: heading stage, V3: anthesis and silking stage, V4: grain-filling stage, V5: mature stage) and peanut (S1: seedling stage, S2: flowering stage, S3: podding stage, S4: mature stage) were sampled during the main growth stages. Three plants were selected in each intercropping plot from the side row (IM for maize, IP for peanut), middle row (MIM for maize, MIP for peanut) and from the sole cropped plots (SM for maize, SP for peanut).

#### Soil samples

We sampled the maize and peanut soil at the same time as the plants. Samples were collected from the side row (IM, IP) and middle row of the intercropped ridge (MIM, MIP), the shared soil of intercropped maize and peanut (II), and the middle row of the sole cropping ridge (SM, SP) (Fig. 9). Soil samples (three replicates) were collected from around the roots (0–20 cm) of maize and peanut. The nitrogen content (TN) of the soil was determined after air drying and sieving. The soil enzymes were measured at the flowering stage of peanut in 2019.

Rhizosphere soil was collected during the flowering stages of peanut in 2019. The shared soil of intercropped maize and peanut (II), the middle row of the sole cropping ridge (SM, SP), the shared soil of the sole cropping (SIM, SIP), and the side row of intercropped ridge (IM, IP) were selected (Fig. 9). The roots were carefully uprooted from the soil and shaken gently to remove loosely attached soil. A sterile brush was used to collect soil from depths of 5–15 cm that adhered firmly to the roots, and then the rhizosphere soil was sieved through a 0.9-mm mesh [48]. Soil samples were separated into two parts: one part was stored at − 80 °C for soil DNA extraction, and the other was stored at 4 °C for analysis of soil physicochemical properties.

### Measurement of nutrients and soil enzyme

The organs of maize (roots, stems, leaves and grains) and peanut (roots, stems, leaves and pods) were heated to 105 °C for 30 min and dried at 80 °C to a constant weight. The total nitrogen (TN) content of the sample was determined by the Kjeldahl method (Kjeltec 8400, Foss, Denmark). Soil samples from different depths in each treatment were sieved and air-dried to determine the soil TN content by the same method.

The activities of the soil enzymes nitrate reductase (NR), peroxidase (POD), protease (Pro) and dehydrogenase (DHO) were measured using an ELISA kit (MLBIO, Shanghai, China). For example, to determine soil NR activity, the kit assayed the soil NR level in the sample; purified soil NR antibody was used to coat the micro titer plate wells, solid-phase antibody was formed, and then NR was added to the wells. The antibody was labelled with HRP, forming an antibody-antigen-enzyme-antibody complex, which was thoroughly washed, followed by the addition of TMB substrate solution. The TMB substrate changed to a blue colour under HRP enzyme-catalysis, the reaction was terminated by the addition of a sulphuric acid solution and the colour change was measured spectrophotometric ally at a wavelength of 450 nm. The concentration of NR in the samples was determined by comparing the OD of the samples with the standard curve.

Spearman correlation analysis was conducted on the soil microbes and soil physiochemical properties in the side row of intercropped plots (IM, IP), the shared soil of intercropped maize and peanut (II), and the middle row of the sole cropping ridge (SM, SP).

### DNA extraction, PCR amplification and high-throughput sequencing

Soil genomic DNA was isolated with the PowerSoil® DNA Isolation kit (MoBio Laboratories, Inc., Carlsbad, CA, USA) according to the manufacturer’s protocols.

The 16S rRNA was amplified for each sample with primer sets of 27F (5′-AGRGTTTGATYNTGGCTCAG-3′) and 1492R (5′-TASGGHTACCTTGTTASGACTT-3′) with adapter sequences and barcode sequences. The ITS was amplified for each sample with primer sets of ITS9 munngs (FCTTGGTCATTTAGAGGAAGTAA) and ITS4 ngsUni (RTCCTCCGCTTATTGATATGC) with adapter seq-uences and barcode sequences. PCR was performed as follows: an initial denatureation at 95 °C for 5 min, followed by 95 °C for 30 s, 50 °C for 30 s and 72 °C f-or 1 min/1 kb then 72 °C for 7 min, for 25–30 cycles. After the electrophoretic r-esults were obtained, all PCR products were quantified by ImageJ software (version 1.4.3.67). After quantification, the samples were mixed according to the required output and fragment size of each sample, and were then recovered and purified with 0.8x magnetic beads to form a sequencing library (SMRT Bell), and the library was subjected to quality inspection [49, 50].

The qualified libraries were sequenced with the PacBio sequencing platform, and the SMRT cell method was used to sequence marker genes at Biomarker Technologies Co., Ltd., Beijing, China. To obtain raw tags, paired-end reads were merged by FLASH (version 1.2.11 http://ccb.jhu.edu/software/FLASH/) [51]. Tags with an average quality score < 20 in a 50 bp sliding window were truncated using Trimmomatic (version 0.33) [52], and tags shorter than 350 bp were removed. We identified possible chimeras by employing UCHIME (version 4.2) [53], and high-quality tags sequences were obtained.

### Statistical and bioinformatics analysis

The plant nutrient content, yield and soil physiochemical properties and diversity indices were tested for differences among wetland restorations with one-way analysis of variance (ANOVA) using SPSS 23.0 for Windows (IBM SPSS Inc., USA). Significance differences were defined at *p* < 0.05. OriginPro version 9.0 (Origin Lab Corporation, Northampton, MA, United States) and R software (version 4.0.3) were used for drawing.

The high-quality sequences were clustered with USEARCH (version 10.0) [54] and tags with similarity ≥97% were regarded as OTUs. Taxonomy was assigned to all OTUs by searching against the Silva databases (Release128, http://www.arb-silva.de) [55], and the UNITE database (Release 8.1, http://unite.ut.ee/index.php) [56], and then were identified down to phylum, class, order, family and genus levels using the Ribosomal Database Project (RDP, version 2.2, http://sourceforge.net/projects/rdpclassifier/), the confidence threshold was 0.8.

Alpha diversity indices referring to community richness (Chao1) and community diversity (Shannon) were calculated by Mothur (version v.1.30, http://www.mothur.org/).

LEfSe [Line Discriminant Analysis (LDA) Effect Size], according to the set screening criteria LDA score > 4, was used to identify significant differences based on biomarkers between different groups [57].

Functional capacity of microbial community and function categorization based on the Kyoto encyclopedia of genes and genomes (KEGG) pathways [58–60]. PICRUSt software (http://kiwi.cs.dal.ca/Software/STAMP) was used to predict the functional gene composition of the samples by comparing the species composition information obtained from 16S sequencing data, to analyse the functional differences between different samples or groups. A paired T-test was performed between different groups and the *p*-value was 0.05 [61]. Fungi Functional Guild (FUN Guild) was used to determine speculate the differential functional gene composition among fungal samples to analyse the functional differences between different samples or groups.

## Supplementary Information

**Additional file 1.**

**Additional file 2.**

**Additional file 3.**

## Data Availability

All raw sequences have been deposited into an NCBI Sequence Read Archive under the accession numbers PRJNA728390 (bacteria) and PRJNA728391 (fungi).

## Abbreviations

SM: Sole maize
SP: Sole peanut
SIM: The shared soil of sole maize
SIP: The shared soil of sole peanut
IM: Intercropped maize
IP: Intercropped peanut
II: The shared soil of intercropped maize and peanut
MIM: The middle row of intercropped maize
MIP: The middle row of intercropped peanut
NR: Nitrate reductase
Pro: Protease
POD: Peroxidase
DHO: Dehydrogenase
TN: Total Nitrogen
OTUs: Operational Taxonomic Units
KEGG: Kyoto Encyclopedia of Genes and Genomes
