# Supplementary material for: Maize/peanut intercropping improves nutrient uptake of side-row maize and system microbial community diversity
Source: BMC Microbiol. 2022 Jan 7;22:14. doi: 10.1186/s12866-021-02425-6 (PMC8740425; doi:10.1186/s12866-021-02425-6)
Supplement: Supplementary file 1 — Additional file 1. [file 12866_2021_2425_MOESM1_ESM.docx]

**BMC Microbiology**

**Supplementary materials**

Maize/peanut intercropping improves nutrient uptake of side-row maize and system microbial community diversity Xinhua Zhao^1^†, Qiqi Dong^1^†, Yi Han^1^, Kezhao Zhang^1^, Xiaolong Shi^1^, Xu Yang^1^, Yang Yuan^1^, Dongying Zhou^1^, Kai Wang^1^, Xiaoguang Wang^1^, Chunji Jiang^1^, Xibo Liu^1^, He Zhang^1^, Zhimeng Zhang^2^, Haiqiu Yu^1^*

^1^ Peanut Research Institute, College of Agronomy, Shenyang Agricultural University, Shenyang 110866, China

^2^ Shandong Peanut Research Institute, Qingdao 266100, Shandong, China

† Xinhua Zhao and Qiqi Dong contributed equally to this work.

* Address Correspondence to Haiqiu Yu: yuhaiqiu@syau.edu.cn

**Table S1 Effect of intercropped maize and peanut on the yield and yield components of maize and peanut in 2018 and 2019**

| **Year** | **Plant** |  | **Ear length** | **Ear diameter** | **Number of grains per spike** | **Weight of 100 grains** | **Yield** |
| --- | --- | --- | --- | --- | --- | --- | --- |
| 2018 | Maize | IM | 20.40a | 17.38a | 760.60a | 191.13a | 15750.08a |
|  |  | MIM | 18.22b | 16.60a | 667.70b | 178.01ab |  |
|  |  | SM | 18.66b | 16.80a | 653.60b | 171.21b | 12083.39b |
|  |  |  | **Number of fruits per plant** | **Number of full fruit per plant** | **100 kernel weight** | **Weight of 100 grains** | **Yield** |
|  | Peanut | IP | 7.00a | 11.64b | 132.17a | 63.61b | 2239.59a |
|  |  | MIP | 8.21a | 13.79b | 135.44a | 63.59a |  |
|  |  | SP | 12.00a | 17.60a | 136.94a | 65.52a | 2989.60a |
| **2019** |  |  | **Ear length** | **Ear diameter** | **Number of grains per spike** | **Weight of 100 grains** | **Yield** |
|  | Maize | IM | 21.90a | 17.10a | 734.20ab | 183.76a | 16250.08a |
|  |  | MIM | 20.46b | 16.90a | 744.40a | 181.95ab |  |
|  |  | SM | 18.20c | 16.20b | 666.00b | 166.03b | 13020.90b |
|  |  |  | **Number of fruits per plant** | **Number of full fruit per plant** | **100 kernel weight** | **Weight of 100 grains** | **Yield** |
|  | Peanut | IP | 6.20a | 9.79a | 129.89b | 64.12 b | 1902.79a |
|  |  | MIP | 8.67a | 10.36a | 133.39a | 64.14 b |  |
|  |  | SP | 7.27a | 8.08a | 134.59a | 66.76a | 1947.93a |

Note: Different letters indicate significant differences at 0.05. SM: sole maize, MIM: the middle row of intercropped maize, IM: intercropped maize, SP: sole peanut, MIP: the middle row of intercropped peanut, IP: intercropped peanut, II: the shared soil of intercropped maize and peanut.

**Table S2** **Changes in the soil TN content under intercropping (mg/kg)**

| Stage | V1 | V2/S1 | V3/S2 | V4/S3 | V5/S4 |
| --- | --- | --- | --- | --- | --- |
| Sample |  |  |  |  |  |
| IM | 7.39±0.15a | 6.63±0.37a | 5.62±0.19a | 4.93±0.54b | 2.31±0.70a |
| IP | 6.98±0.50a | 6.40±0.31a | 7.13±0.22a | 9.67±0.29a | 3.38±0.05a |
| II | 7.26±0.16a | 6.51±0.20a | 6.99±0.52a | 5.07±1.03b | 3.04±0.47a |

Note: Different letters indicate significant differences at 0.05. V1, trumpeting stage; V2, heading stage; V3, anthesis and silking stage; V4, grain-filling stage; V5, mature stage; S1, seedling stage; S2, flowering stage; S3, podding stage; S4, mature stage. IM: intercropped maize, IP: intercropped peanut, II: the shared soil of intercropped maize and peanut.

**Table S3 Changes in soil enzyme activities under intercropping**

| Sample | NR/(U/L) | Pro/(IU/L) | POD/(U/L) | DHO/(IU/L) |
| --- | --- | --- | --- | --- |
| IM | 59.48±5.70a | 195.89±10.83a | 387.91±62.16a | 51.77±8.69a |
| IP | 57.30±1.89a | 193.62±3.32a | 375.83±19.86a | 50.79±1.88a |
| II | 58.76±7.35a | 195.99±2.69a | 376.82±34.86a | 67.23±2.61a |

Note: Different letters indicate significant differences at 0.05. IM: intercropped maize, IP: intercropped peanut, II: the shared soil of intercropped maize and peanut.

**Table S6 The initial soil properties of the experimental field**

| AN | AP | AK | SOC | PH |
| --- | --- | --- | --- | --- |
| 2100.70mg/kg | 27.50mg/kg | 117.90mg/kg | 15170.00mg/kg | 6.50 |

**Table S7 Fertilizer application to** **sole and intercropped models**

| Fertilizer application of maize (kg/hm^2^) | | |  | Fertilizer application of peanut (kg/hm^2^) | | |
| --- | --- | --- | --- | --- | --- | --- |
| N | P_2_O_5_ | K_2_O |  | N | P_2_O_5_ | K_2_O |
| 202.50 | 97.50 | 112.50 |  | 105.00 | 129.00 | 112.50 |

**Table S8 Sowing and harvest dates of maize and peanut**

| Year | Maize | |  | Peanut | |
| --- | --- | --- | --- | --- | --- |
|  | Sowing | Harvest |  | Sowing | Harvest |
| 2018 | 5.15 | 9.27 |  | 5.15 | 9.24 |
| 2019 | 5.12 | 9.25 |  | 5.12 | 9.20 |


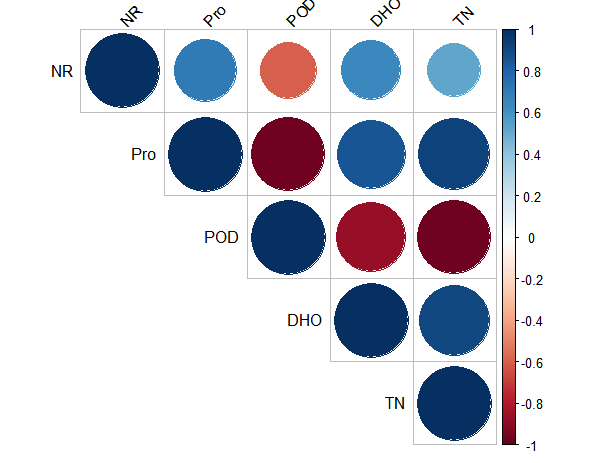


**Figure S1. Correlation analysis of soil enzyme activity and soil TN**


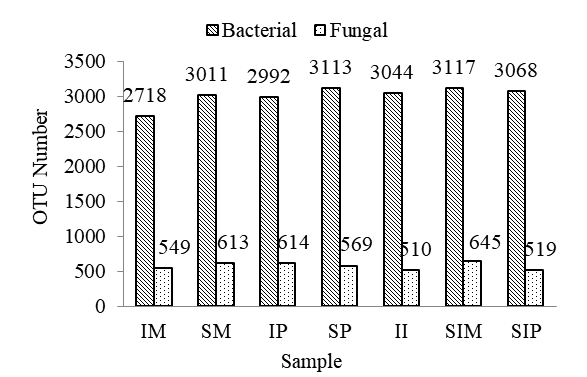


**Figure S2. Number of observed operational taxonomic units (OTUs) of rhizosphere soil**

SM: sole maize, SIM: the shared soil of sole maize, IM: intercropped maize, SP: sole peanut, SIP: the shared soil of sole peanut, IP: intercropped peanut, II: the shared soil of intercropped maize and peanut.


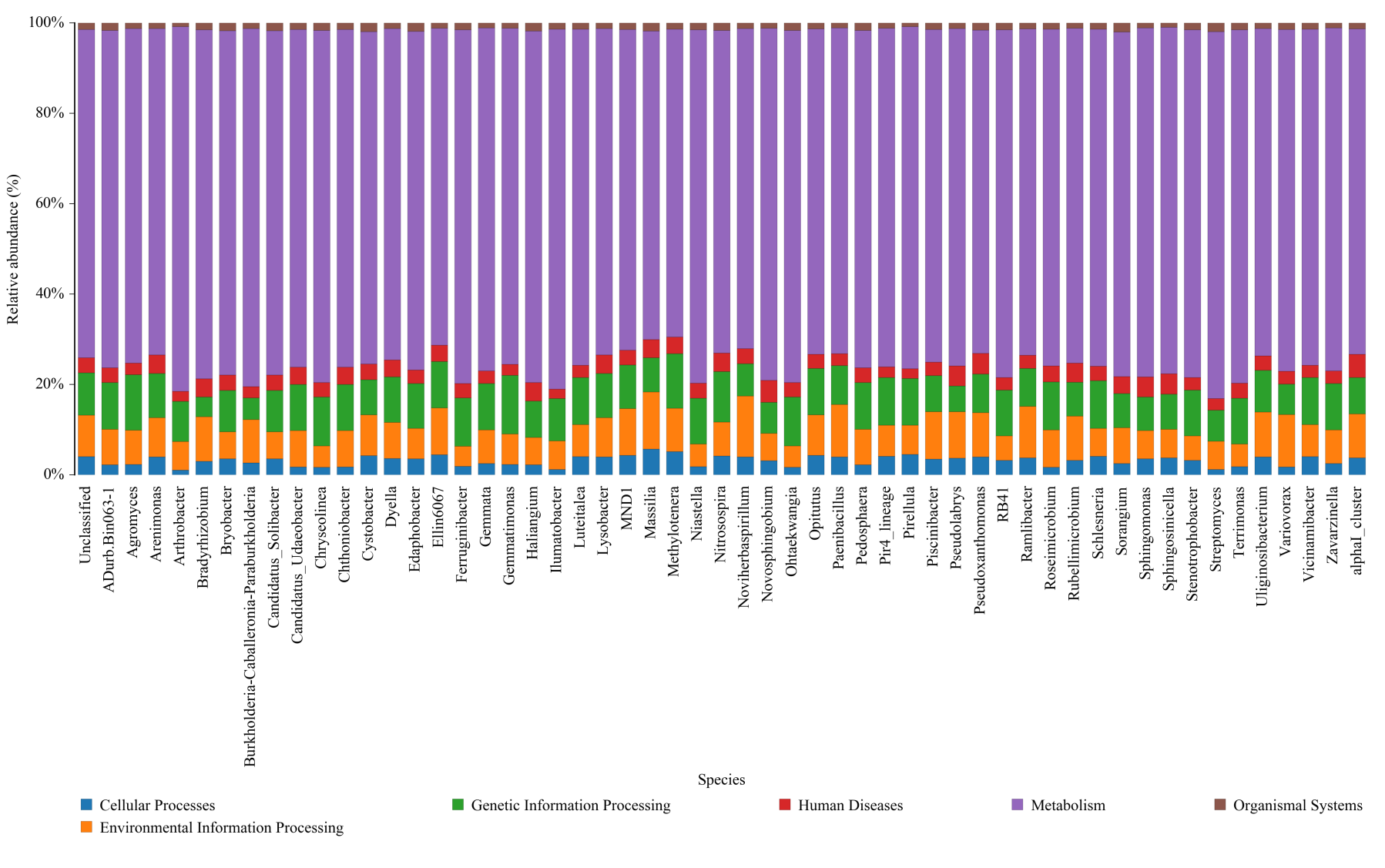


**Figure S3 KEGG functional annotation of the first level of the bacterial community**
